# Particular Account of the Illness and Death of George Washington

**Published:** 1800-05

**Authors:** James Craik, Elisha C. Dick

**Affiliations:** Attending Physician; Consulting Physician


*The following is a Copy of the Physicians' Report upon the Death of General Washington; which Report is referred to by Dr. Reid in his Observations contained in this Number, p. 473.*

ALEXANDRIA, (VIRG.) DEC. 21, 1799.

### Particular Account of the late Illness and Death of GEORGE WASHINGTON.

Some time in the night of Friday, the 10th instant, having been exposed to a rain on the preceding day, General Washington was attacked with an inflammatory affection of the upper part of the wind pipe, called, in technical language, Cynanche Trachealis. The disease commenced with a violent ague, accompanied with some pain in the upper and fore part of the throat, a sense of stricture in the same part, a cough, and a difficult rather than a painful deglutition, which were soon succeeded by fever and a quick and laborious respiration. The necessity of blood-letting suggesting itself to the General, he procured a bleeder in the neighbourhood, who took from his arm, in the night, twelve or fourteen ounces of blood. He could not by any means be prevailed on by the family to send for the attending physician till the following morning, who arrived at Mount Vernon, at about eleven o'clock on Saturday. Discovering the case to be highly alarming, and foreseeing the fatal tal tal tendency of the diſeaſe, two conſulting phyſicians were immediately ſent for, who arrived, one at half after three, and the other at four o'clock in the afternoon: In the mean time were employed two pretty copious bleedings, a bliſter was applied to the part affected, two moderate doſes of calomel were given, and an injection was adminiſtered, which operated on the lower inteſtines, but all without any perceptible advantage, the reſpiration becoming ſtill more difficult and diſtreſſing. Upon the arrival of the firſt of the conſulting phyſicians, it was agreed, as there were yet no ſigns of accumulation in the bronchial veſſels of the lungs, to try the reſult of another bleeding, when about thirty-two ounces were drawn, without the ſmalleſt apparent alleviation of the diſeaſe. Vapours of vinegar and water were frequently inhaled, ten grains of calomel were given, ſucceeded by repeated doſes of emetic tartar, amounting in all to five or ſix grains, with no other effect than a copious diſcharge from the bowels. The powers of life ſeemed now manifeſtly yielding to the force of the diſorder; bliſters were applied to the extremities, together with a cataplaſm of bran and vinegar to the throat. Speaking, which was painful from the beginning, now became almoſt impracticable; reſpiration grew more and more contracted and imperfect, till half after eleven on Saturday night, retaining the full poſſeſſion of his intellect—when he expired without a ſtruggle.

He was fully impreſſed at the beginning of his complaint, as well as through every ſucceeding ſtage of it, that its concluſion would be mortal; ſubmitting to the ſeveral exertions made for his recovery, rather as a duty, than from any expectation of their efficacy. He conſidered the operations of death upon his ſyſtem as coeval with the diſeaſe; and ſeveral hours before his death, after repeated efforts to be underſtood, ſucceeded in expreſſing a deſire that he might be permitted to die without further interruption.

During the ſhort period of his illneſs, he economized his time, in the arrangement of ſuch few concerns as required his attention, with the utmoſt ſerenity; and anticipated his approaching diſſolution with every demonſtration of that equanimity for which his whole life has been ſo uniformly and ſingularly conſpicuous.

JAMES CRAIK, Attending Phyſician.

ELISHA C. DICK, Conſulting Phyſician.

